# Bis(acetato-κ^2^ *O*,*O*′)[2,6-bis­(1*H*-pyrazol-3-yl-κ*N* ^2^)pyridine-κ*N*]manganese(II)

**DOI:** 10.1107/S1600536811010506

**Published:** 2011-03-26

**Authors:** Fan Yu, Bao Li

**Affiliations:** a School of Chemistry and Environmental Engineering, Jianghan University, Wuhan 430056, People’s Republic of China; b Department of Chemistry and Chemical Engineering, Huazhong University of Science and Technology, Wuhan 430074, People’s Republic of China

## Abstract

In the title complex, [Mn(CH_3_CO_2_)_2_(C_11_H_9_N_5_)], the Mn^II^ atom is coordinated by the pyridine N atom and two pyrazole N atoms from a 2,6-bis­(pyrazol-3-yl)pyridine ligand and four O atoms from two bidentate acetate ligands. The complex mol­ecules are linked by inter­molecular N—H⋯O hydrogen bonds into a chain along [010]. π–π inter­actions between the pyridine rings and between the pyrazole rings [centroid–centroid distances = 3.772 (2) and 3.546 (2) Å] connect the chains.

## Related literature

For a related structure, see: Rich *et al.* (2010 ▶).
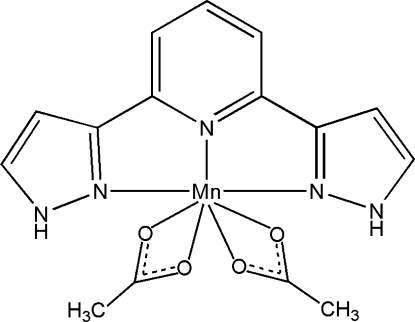


## Experimental

### 

#### Crystal data

[Mn(C_2_H_3_O_2_)_2_(C_11_H_9_N_5_)]
*M* *_r_* = 384.26
Triclinic, 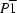

*a* = 8.2386 (16) Å
*b* = 9.4324 (19) Å
*c* = 11.081 (2) Å
α = 98.32 (3)°
β = 95.01 (3)°
γ = 106.11 (3)°
*V* = 811.2 (3) Å^3^
*Z* = 2
Mo *K*α radiation
μ = 0.85 mm^−1^
*T* = 293 K
0.30 × 0.20 × 0.20 mm

#### Data collection

Rigaku R-AXIS RAPID diffractometer
Absorption correction: multi-scan (*ABSCOR*; Higashi, 1995 ▶) *T*_min_ = 0.816, *T*_max_ = 0.849
5563 measured reflections
3001 independent reflections
2386 reflections with *I* > 2σ(*I*)
*R*_int_ = 0.030

#### Refinement

*R*[*F* ^2^ > 2σ(*F* ^2^)] = 0.034
*wR*(*F* ^2^) = 0.117
*S* = 1.10
3001 reflections
226 parameters
H-atom parameters constrained
Δρ_max_ = 0.51 e Å^−3^
Δρ_min_ = −0.43 e Å^−3^

### 

Data collection: *RAPID-AUTO* (Rigaku, 1998 ▶); cell refinement: *RAPID-AUTO*; data reduction: *CrystalStructure* (Rigaku/MSC, 2002 ▶); program(s) used to solve structure: *SHELXS97* (Sheldrick, 2008 ▶); program(s) used to refine structure: *SHELXL97* (Sheldrick, 2008 ▶); molecular graphics: *SHELXTL* (Sheldrick, 2008 ▶) and *Mercury* (Macrae *et al.*, 2006 ▶); software used to prepare material for publication: *SHELXTL*.

## Supplementary Material

Crystal structure: contains datablocks I, global. DOI: 10.1107/S1600536811010506/hy2414sup1.cif

Structure factors: contains datablocks I. DOI: 10.1107/S1600536811010506/hy2414Isup2.hkl

Additional supplementary materials:  crystallographic information; 3D view; checkCIF report

## Figures and Tables

**Table 1:** Selected bond lengths (Å)

Mn1—O1	2.480 (2)
Mn1—O2	2.192 (2)
Mn1—O3	2.596 (2)
Mn1—O4	2.160 (2)
Mn1—N2	2.262 (2)
Mn1—N3	2.235 (2)
Mn1—N4	2.270 (2)

**Table 2:** Hydrogen-bond geometry (Å, °)

*D*—H⋯*A*	*D*—H	H⋯*A*	*D*⋯*A*	*D*—H⋯*A*
N1—H1*B*⋯O4^i^	0.86	1.91	2.751 (3)	165
N5—H5*B*⋯O2^ii^	0.86	1.85	2.712 (3)	180

Symmetry codes: (i) 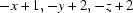; (ii) 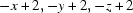.

**Fig. 1.**
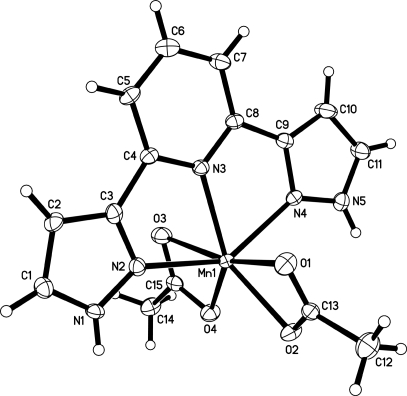
Molecular structure of the title compound. Displacement ellipsoids are drawn at the 50% probability level.

**Fig. 2.**
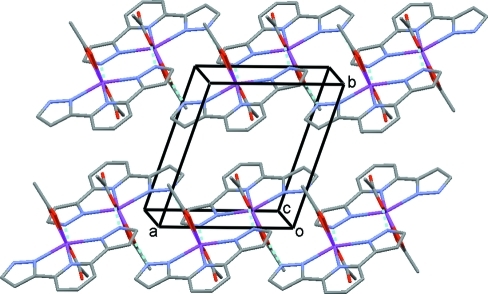
Crystal packing of the title compound, showing the chain structures along the b axis. Dashed lines denote hydrogen bonds. H atoms have been omitted for clarity.

## supplementary crystallographic information

### Experimental

2,6-Bis(pyrazol-3-yl)pyridine (0.1 mmol) was dissolved in methanol (2.5 ml) with 0.2 mmol of trimethylamine. Mn(OAc)_2_ (0.2 mmol) in methanol (2.5 ml) was added into the resulting solution. After stirring at room temperature for 1 h, the resulting yellow solution was put into a tube layered with aether. Yellow crystals were obtained in three days.

### Refinement

H atoms were positioned geometrically and refined as riding atoms, with C—H = 0.93 (aromatic), 0.96 (CH_3_) and N—H = 0.86 Å and with *U*_iso_(H) = 1.2(1.5 for methyl)*U*_eq_(C, N).

### Crystal data

**Table:**

[Mn(C_2_H_3_O_2_)_2_(C_11_H_9_N_5_)]	*Z* = 2
*M**_r_* = 384.26	*F*(000) = 394
Triclinic, *P*1	*D*_x_ = 1.573 Mg m^−^^3^
Hall symbol: -P 1	Mo *K*α radiation, λ = 0.71073 Å
*a* = 8.2386 (16) Å	Cell parameters from 2386 reflections
*b* = 9.4324 (19) Å	θ = 6.1–54.9°
*c* = 11.081 (2) Å	µ = 0.85 mm^−^^1^
α = 98.32 (3)°	*T* = 293 K
β = 95.01 (3)°	Block, yellow
γ = 106.11 (3)°	0.30 × 0.20 × 0.20 mm
*V* = 811.2 (3) Å^3^	

### Data collection

**Table:**

Rigaku R-AXIS RAPID diffractometer	3001 independent reflections
Radiation source: rotation anode	2386 reflections with *I* > 2σ(*I*)
graphite	*R*_int_ = 0.030
ω scans	θ_max_ = 26.0°, θ_min_ = 3.4°
Absorption correction: multi-scan (*ABSCOR*; Higashi, 1995)	*h* = −10→10
*T*_min_ = 0.816, *T*_max_ = 0.849	*k* = −11→9
5563 measured reflections	*l* = −13→13

### Refinement

**Table:**

Refinement on *F*^2^	Primary atom site location: structure-invariant direct methods
Least-squares matrix: full	Secondary atom site location: difference Fourier map
*R*[*F*^2^ > 2σ(*F*^2^)] = 0.034	Hydrogen site location: inferred from neighbouring sites
*wR*(*F*^2^) = 0.117	H-atom parameters constrained
*S* = 1.10	*w* = 1/[σ^2^(*F*_o_^2^) + (0.0565*P*)^2^] where *P* = (*F*_o_^2^ + 2*F*_c_^2^)/3
3001 reflections	(Δ/σ)_max_ = 0.001
226 parameters	Δρ_max_ = 0.51 e Å^−^^3^
0 restraints	Δρ_min_ = −0.43 e Å^−^^3^

### Fractional atomic coordinates and isotropic or equivalent isotropic displacement parameters (Å^2^)

**Table:**

	*x*	*y*	*z*	*U*_iso_*/*U*_eq_	
Mn1	0.67466 (5)	0.89985 (4)	0.81715 (4)	0.01323 (17)	
N1	0.3285 (3)	1.0204 (2)	0.7643 (2)	0.0155 (5)	
H1B	0.3327	1.0677	0.8375	0.019*	
N2	0.4400 (3)	0.9459 (2)	0.7303 (2)	0.0155 (5)	
N3	0.6132 (3)	0.7810 (2)	0.6217 (2)	0.0131 (5)	
N4	0.8712 (3)	0.7781 (2)	0.7766 (2)	0.0148 (5)	
N5	1.0113 (3)	0.7595 (3)	0.8392 (2)	0.0179 (5)	
H5B	1.0537	0.8029	0.9135	0.021*	
C1	0.2104 (4)	1.0115 (3)	0.6700 (3)	0.0187 (6)	
H1A	0.1217	1.0545	0.6732	0.022*	
C2	0.2431 (4)	0.9274 (3)	0.5670 (3)	0.0169 (6)	
H2A	0.1842	0.9025	0.4877	0.020*	
C3	0.3877 (3)	0.8887 (3)	0.6116 (3)	0.0156 (6)	
C4	0.4818 (3)	0.7946 (3)	0.5476 (2)	0.0135 (6)	
C5	0.4382 (4)	0.7223 (3)	0.4265 (2)	0.0181 (6)	
H5A	0.3462	0.7324	0.3771	0.022*	
C6	0.5374 (4)	0.6335 (3)	0.3808 (3)	0.0225 (7)	
H6A	0.5109	0.5824	0.2999	0.027*	
C7	0.6738 (4)	0.6213 (3)	0.4548 (3)	0.0204 (7)	
H7A	0.7413	0.5638	0.4244	0.025*	
C8	0.7087 (4)	0.6962 (3)	0.5750 (2)	0.0145 (6)	
C9	0.8480 (4)	0.6925 (3)	0.6657 (3)	0.0158 (6)	
C10	0.9715 (4)	0.6183 (4)	0.6587 (3)	0.0252 (7)	
H10A	0.9825	0.5508	0.5922	0.030*	
C11	1.0740 (4)	0.6645 (3)	0.7698 (3)	0.0213 (7)	
H11A	1.1696	0.6353	0.7926	0.026*	
C12	1.0613 (4)	1.3063 (3)	0.8677 (3)	0.0306 (8)	
H12A	1.0919	1.3407	0.7931	0.046*	
H12B	1.1568	1.2860	0.9100	0.046*	
H12C	1.0294	1.3823	0.9194	0.046*	
C13	0.9129 (4)	1.1649 (3)	0.8373 (3)	0.0166 (6)	
O1	0.8498 (3)	1.1097 (2)	0.7299 (2)	0.0253 (5)	
O2	0.8561 (3)	1.1034 (2)	0.92644 (17)	0.0196 (5)	
C14	0.3975 (4)	0.6485 (3)	1.0567 (3)	0.0228 (7)	
H14A	0.3170	0.5539	1.0197	0.034*	
H14B	0.3380	0.7142	1.0935	0.034*	
H14C	0.4766	0.6332	1.1188	0.034*	
C15	0.4932 (4)	0.7179 (3)	0.9591 (3)	0.0153 (6)	
O3	0.4611 (3)	0.6531 (2)	0.85024 (18)	0.0227 (5)	
O4	0.6077 (3)	0.8442 (2)	0.99224 (18)	0.0195 (5)	

### Atomic displacement parameters (Å^2^)

**Table:**

	*U*^11^	*U*^22^	*U*^33^	*U*^12^	*U*^13^	*U*^23^
Mn1	0.0131 (3)	0.0136 (3)	0.0113 (2)	0.00332 (17)	0.00028 (18)	−0.00105 (16)
N1	0.0176 (13)	0.0184 (12)	0.0118 (12)	0.0079 (10)	0.0042 (10)	0.0006 (9)
N2	0.0136 (13)	0.0139 (12)	0.0192 (13)	0.0045 (10)	0.0024 (10)	0.0029 (10)
N3	0.0139 (12)	0.0139 (12)	0.0114 (12)	0.0035 (10)	0.0023 (10)	0.0024 (9)
N4	0.0146 (12)	0.0180 (12)	0.0110 (12)	0.0050 (10)	−0.0001 (10)	0.0011 (9)
N5	0.0161 (13)	0.0185 (13)	0.0185 (13)	0.0047 (10)	−0.0002 (10)	0.0037 (10)
C1	0.0177 (16)	0.0223 (15)	0.0179 (15)	0.0082 (13)	0.0028 (13)	0.0052 (12)
C2	0.0176 (15)	0.0212 (15)	0.0110 (14)	0.0052 (12)	−0.0009 (12)	0.0031 (11)
C3	0.0128 (14)	0.0119 (14)	0.0216 (16)	0.0020 (11)	0.0035 (12)	0.0039 (11)
C4	0.0158 (15)	0.0142 (14)	0.0099 (14)	0.0021 (11)	0.0026 (12)	0.0038 (11)
C5	0.0221 (16)	0.0204 (15)	0.0103 (14)	0.0053 (13)	−0.0028 (12)	0.0031 (11)
C6	0.0296 (18)	0.0196 (16)	0.0186 (16)	0.0085 (14)	0.0045 (14)	0.0012 (12)
C7	0.0250 (17)	0.0215 (16)	0.0172 (16)	0.0113 (13)	0.0050 (13)	0.0010 (12)
C8	0.0176 (15)	0.0153 (14)	0.0106 (14)	0.0047 (12)	0.0021 (12)	0.0026 (11)
C9	0.0164 (15)	0.0170 (14)	0.0143 (15)	0.0049 (12)	0.0041 (12)	0.0022 (11)
C10	0.0300 (19)	0.0326 (18)	0.0166 (16)	0.0195 (15)	0.0017 (14)	−0.0033 (13)
C11	0.0212 (17)	0.0286 (17)	0.0176 (16)	0.0141 (14)	0.0006 (13)	0.0032 (13)
C12	0.0286 (19)	0.0187 (16)	0.039 (2)	−0.0018 (14)	0.0063 (16)	0.0045 (14)
C13	0.0159 (15)	0.0148 (14)	0.0201 (16)	0.0086 (12)	0.0002 (13)	0.0000 (12)
O1	0.0234 (12)	0.0237 (12)	0.0262 (12)	0.0056 (10)	0.0001 (10)	0.0003 (9)
O2	0.0236 (11)	0.0167 (10)	0.0145 (11)	0.0011 (9)	0.0007 (9)	0.0004 (8)
C14	0.0275 (17)	0.0185 (15)	0.0200 (16)	0.0025 (13)	0.0024 (14)	0.0044 (12)
C15	0.0186 (15)	0.0174 (14)	0.0144 (15)	0.0127 (12)	0.0007 (12)	0.0041 (11)
O3	0.0272 (12)	0.0271 (12)	0.0158 (11)	0.0123 (10)	0.0026 (9)	0.0017 (9)
O4	0.0219 (11)	0.0206 (11)	0.0147 (11)	0.0035 (9)	0.0039 (9)	0.0034 (8)

### Geometric parameters (Å, °)

**Table:**

Mn1—O1	2.480 (2)	C5—C6	1.399 (4)
Mn1—O2	2.192 (2)	C5—H5A	0.9300
Mn1—O3	2.596 (2)	C6—C7	1.373 (4)
Mn1—O4	2.160 (2)	C6—H6A	0.9300
Mn1—N2	2.262 (2)	C7—C8	1.380 (4)
Mn1—N3	2.235 (2)	C7—H7A	0.9300
Mn1—N4	2.270 (2)	C8—C9	1.469 (4)
N1—C1	1.341 (4)	C9—C10	1.387 (4)
N1—N2	1.349 (3)	C10—C11	1.370 (4)
N1—H1B	0.8600	C10—H10A	0.9300
N2—C3	1.332 (4)	C11—H11A	0.9300
N3—C4	1.347 (3)	C12—C13	1.510 (4)
N3—C8	1.353 (3)	C12—H12A	0.9600
N4—C9	1.336 (3)	C12—H12B	0.9600
N4—N5	1.361 (3)	C12—H12C	0.9600
N5—C11	1.338 (4)	C13—O1	1.233 (3)
N5—H5B	0.8600	C13—O2	1.275 (3)
C1—C2	1.383 (4)	C14—C15	1.512 (4)
C1—H1A	0.9300	C14—H14A	0.9600
C2—C3	1.410 (4)	C14—H14B	0.9600
C2—H2A	0.9300	C14—H14C	0.9600
C3—C4	1.478 (4)	C15—O3	1.241 (3)
C4—C5	1.382 (4)	C15—O4	1.278 (3)
			
O4—Mn1—O2	85.32 (8)	N3—C4—C5	122.6 (2)
O4—Mn1—N3	135.59 (8)	N3—C4—C3	112.7 (2)
O2—Mn1—N3	138.38 (8)	C5—C4—C3	124.6 (3)
O4—Mn1—N2	103.71 (8)	C4—C5—C6	117.6 (3)
O2—Mn1—N2	111.90 (8)	C4—C5—H5A	121.2
N3—Mn1—N2	71.44 (8)	C6—C5—H5A	121.2
O4—Mn1—N4	103.09 (8)	C7—C6—C5	120.3 (3)
O2—Mn1—N4	95.79 (8)	C7—C6—H6A	119.8
N3—Mn1—N4	71.19 (8)	C5—C6—H6A	119.8
N2—Mn1—N4	142.63 (8)	C6—C7—C8	118.8 (3)
O4—Mn1—O1	140.27 (8)	C6—C7—H7A	120.6
O2—Mn1—O1	55.33 (7)	C8—C7—H7A	120.6
N3—Mn1—O1	84.13 (8)	N3—C8—C7	122.0 (3)
N2—Mn1—O1	88.11 (8)	N3—C8—C9	112.8 (2)
N4—Mn1—O1	87.66 (8)	C7—C8—C9	125.2 (3)
O4—Mn1—O3	54.13 (7)	N4—C9—C10	109.9 (3)
O2—Mn1—O3	139.25 (7)	N4—C9—C8	117.9 (2)
N3—Mn1—O3	81.62 (7)	C10—C9—C8	132.1 (3)
N2—Mn1—O3	84.46 (7)	C11—C10—C9	106.1 (3)
N4—Mn1—O3	90.63 (7)	C11—C10—H10A	127.0
O1—Mn1—O3	165.39 (6)	C9—C10—H10A	127.0
C1—N1—N2	111.6 (2)	N5—C11—C10	107.3 (3)
C1—N1—H1B	124.2	N5—C11—H11A	126.4
N2—N1—H1B	124.2	C10—C11—H11A	126.4
C3—N2—N1	104.9 (2)	C13—C12—H12A	109.5
C3—N2—Mn1	117.16 (17)	C13—C12—H12B	109.5
N1—N2—Mn1	137.93 (18)	H12A—C12—H12B	109.5
C4—N3—C8	118.8 (2)	C13—C12—H12C	109.5
C4—N3—Mn1	120.55 (17)	H12A—C12—H12C	109.5
C8—N3—Mn1	120.66 (18)	H12B—C12—H12C	109.5
C9—N4—N5	105.9 (2)	O1—C13—O2	121.1 (3)
C9—N4—Mn1	117.09 (18)	O1—C13—C12	121.2 (3)
N5—N4—Mn1	136.93 (17)	O2—C13—C12	117.8 (3)
C11—N5—N4	110.9 (2)	C13—O1—Mn1	85.49 (18)
C11—N5—H5B	124.6	C13—O2—Mn1	97.79 (17)
N4—N5—H5B	124.6	C15—C14—H14A	109.5
N1—C1—C2	108.3 (2)	C15—C14—H14B	109.5
N1—C1—H1A	125.8	H14A—C14—H14B	109.5
C2—C1—H1A	125.8	C15—C14—H14C	109.5
C1—C2—C3	103.0 (3)	H14A—C14—H14C	109.5
C1—C2—H2A	128.5	H14B—C14—H14C	109.5
C3—C2—H2A	128.5	O3—C15—O4	121.3 (3)
N2—C3—C2	112.1 (2)	O3—C15—C14	120.6 (3)
N2—C3—C4	118.0 (2)	O4—C15—C14	118.1 (2)
C2—C3—C4	129.8 (3)	C15—O4—Mn1	101.76 (17)

### Hydrogen-bond geometry (Å, °)

**Table:**

*D*—H···*A*	*D*—H	H···*A*	*D*···*A*	*D*—H···*A*
N1—H1B···O4^i^	0.86	1.91	2.751 (3)	165
N5—H5B···O2^ii^	0.86	1.85	2.712 (3)	180

Symmetry codes: (i) −*x*+1, −*y*+2, −*z*+2; (ii) −*x*+2, −*y*+2, −*z*+2.
